# Biallelic variants in IBA57 with multiple mitochondrial dysfunction syndrome 3

**DOI:** 10.3389/fgene.2026.1815601

**Published:** 2026-07-20

**Authors:** Yijuan Huang, Chenyu Gou, Yuanqiu Chen, Qihao Pan, Tongtong Chen, Zhiqiang Zhang, Xiaohong Wang, Si Li, Yufei Han, Weiying Jiang, Yu Gao

**Affiliations:** 1 Department of Obstetrics and Gynecology, The Sixth Affiliated Hospital, Sun Yat-sen University, Guangzhou, China; 2 Guangzhou (Huangpu) Zhongliu Biomedical Innovation Center, Guangzhou, China; 3 School of Medicine, The Chinese University of Hong Kong, Shenzhen, China; 4 Warshel Institute for Computational Biology, School of Medicine, The Chinese University of Hong Kong, Shenzhen, China; 5 Reproductive Medicine Center, The Sixth Affiliated Hospital of Sun Yat-sen University, Guangzhou, China; 6 Guangdong Engineering Technology Research Center of Fertility Preservation, Guangzhou, China; 7 Shanghai Yikon Medical Laboratory Co., Ltd, Shanghai, China; 8 Department of Medical Genetics and Bioinformatics, Zhongshan Medical School, Sun Yat-sen University, Guangzhou, China

**Keywords:** amniotic fluid cells, citric acid cycle, IBA57, MMDS3, PI3K-Akt

## Abstract

**Background:**

Multiple mitochondrial dysfunction syndrome type 3 (MMDS3; OMIM #615330) is a rare autosomal recessive disorder caused by mutations in *IBA57*. Its complex clinical presentation and molecular pathogenesis remain incompletely understood.

**Methods:**

The study included comprehensive clinical evaluation, *IBA5*7 genetic testing, Western Blotting for protein expression, and transcriptomic and metabolomic analyses of amniotic fluid cells.

**Results:**

The proband presented with typical MMDS3 features, and both affected siblings carried compound heterozygous *IBA57* missense mutations (c.310G>T and c.826C>T) leading to reduced IBA57 protein expression. RNA-seq revealed transcriptional dysregulation of the PI3K-Akt signaling pathway, and metabolomics demonstrated TCA cycle disturbances in amniocytes. Respiratory chain enzyme assays showed a selective deficiency of complex II activity in fetal liver.

**Conclusion:**

The compound heterozygous *IBA57* mutations c.310G>T and c.826C>T lead to reduced IBA57 protein expression, selective impairment of respiratory chain complex II, and transcriptional dysregulation of the PI3K-Akt pathway, together contributing to the MMDS3 phenotype in the proband and the affected fetus.

## 1 Introduction

Multiple Mitochondrial Dysfunction Syndromes (MMDS) are a group of mitochondrial disorders involving multiple genes, predominantly characterized by mitochondrial iron-sulfur cluster assembly defects (Lill, 2020). This leads to high phenotypic variability, including white matter abnormalities, generalized muscle weakness, lactic acidosis, hyperglycinemia, cavitating leukoencephalopathy, respiratory failure, and early mortality due to impaired energy metabolism in various systems. The *IBA57* gene encodes a late-acting assembly factor in the mitochondrial Fe-S cluster biogenesis pathway. While early-acting components (e.g., the ISCU scaffold complex) are responsible for the *de novo* synthesis of labile [2Fe-2S] clusters, IBA57 functions downstream to mediate the maturation of these clusters into stable [4Fe-4S] forms and facilitates their specific insertion into recipient apoproteins (Gourdoupis S et al., 2018). A variety of deleterious *IBA57* mutations have been identified in multiple individuals (Camponeschi F et al., 2022)now commonly subsumed under autosomal recessive multiple mitochondrial dysfunctions syndrome 3 (MMDS3:OMIM#615330).

In this study, we report two affected siblings from a Chinese family presenting with MMDS3: an infant and a fetus, with the aim of improving early recognition and contributing to the understanding of the pathogenesis of this disorder. Prenatal diagnosis confirmed the same compound heterozygous missense mutations in *IBA57* in the second fetus. To further explore the functional consequences of the identified variants, we performed transcriptomic (RNA-seq) and metabolomic analyses on amniocytes, and conducted a comprehensive literature review of MMDS3.

## 2 Materials and methods

### 2.1 Ethical considerations and participants

This study was approved by the Human Ethics Committees of Sixth Affiliated Hospital, Sun Yat-Sen University. Written informed consent for participation in the study and for publication of clinical data and images was obtained from the legal guardians of all participants.

### 2.2 Whole-exome sequencing

Genomic DNA was extracted from the proband and family members using the Maxwell RSC Blood DNA kit (Magen, China). Whole-exome capture was performed with the IDT xGen Exome Research Panel v2, and 150-bp paired-end sequencing was carried out on an Illumina NovaSeq 6000 platform. Bioinformatics analyses were performed following standard pipelines, and the detailed methods for mitochondrial genome analysis and variant classification are provided in the Supplementary Methods.

#### 2.2.1 Chromosomal microarray analysis and classification

Chromosomal microarray analysis was performed using the Illumina Infinium OmniZhongHua-8 v1.4 BeadChip according to the manufacturer’s instructions. Data were analyzed with GenomeStudio 2.0 software, and copy-number variants were classified following the ACMG/ClinGen recommendations, with supporting information from DECIPHER, OMIM, and gnomAD.

### 2.3 Amniocyte culture

Amniotic fluid samples were obtained by amniocentesis at 21 weeks of gestation at the Department of Obstetrics and Gynecology. The case sample was collected from the affected fetus in the family’s second pregnancy (the proband’s sibling). Three control amniotic fluid samples were obtained from pregnant women undergoing amniocentesis for advanced maternal age, with normal amniotic fluid results and confirmed normal fetal development. 20 mL of amniotic fluid was collected from patient and centrifuged (3000 rpm, 5 min, 4 °C). The cell-free amniotic fluid was then separated for TORCH assays, whereas amniocytes were cultured using standard methods for gene analyse (Luo HY et al., 1984; Vamos, 1984). Amniocytes were cultured in Gibco® AmnioMAX™ C-100 Complete Medium (GIBCO BRL, USA)According to the routine protocols.

### 2.4 Western Blotting (WB) analysis

The lysis buffer used in our experiments, which is comprised of RIPA lysis solution and tris-triton among other components, is utilized to lyse amniotic fluid cells at 4 °C. Subsequently, centrifugation is performed to remove the supernatant. Proteins were solubilized and subsequently separated by tricine SDS PAGE. For structural studies, amniocyte were processed as described and 40–80 ug of proteins were loaded on linear 5%–12% gradient gel for first dimensional blue native gel electrophoresis (BNGE). BNGE was later, either subjected to in-gel activity assay (IGA) processed for Western Blotting (WB) analysis. WB of either BNGE or SDS-PAGE was performed by transferring proteins onto polyvinylidene difluoride (PVDF) membrane and probed with specific antibodies. Reactive bands were detected using Lite Ablot Extend Long Lasting Chemiluminescent Substrate (Euroclone, Pero (Mi), Italy). Densitometry analysis was performed using Quantity One software (BioRad, Hercules, CA,USA). Anti-C1orf69/IBA57 antibody [EPR11758(2)](Ab180161), VDAC(D73D12) Rabbit mAb #4661.

### 2.5 Histology studies

A muscle was about 0.8 × 0.5 × 0.7 cm obtained from the left gluteus maximus of the fetal as part of routine diagnostic procedures. Liquid nitrogen cross sections of gluteus maximus muscle biopsies were used for histological and histochemical studies, and processed according to standard techniques. The electron microscopy (EM)-based ultra-structural studies were conducted on the index patient’s muscle biopsy samples. The patient’s tissue been sent to third-party detection institution(Department of Neurology Laboratory, Nanfang Hospital, Southern Medical University, Guangzhou Kinyu Pathology Department) to processed the diagnosis.

### 2.6 RNA sequencing

RNA sequencing were analyzed in the patient’s amniocytes that were cultured samples(*IBA57*-mut) and normal controls(Wild-type). A total amount of 1 µg RNA per sample was used as input material for the RNA sample preparations. Sequencing libraries were generated using NEBNext ® Ultra TM RNA Library Prep Kit for Illumina ® (CatalogE7530L, NEB, USA) following manufacturer’s recommendations and index codes were added to attribute sequences to each sample. Briefly, mRNA was purified from total RNA using oligo d(T) 25 magnetic beads. Fragmentation was carried out using divalent cations under elevated temperature in NEB Next First Strand Synthesis Reaction Buffer(5X). First strand cDNA was synthesized using random primer and ProtoScript ® II Reverse Transcriptase. Second strand cDNA synthesis was subsequently performed using DNA Polymerase I and RNase H. Remaining overhangs were converted into blunt ends via exonuclease/polymerase activities. After adenylation of 3′ ends of DNA fragments, single-index adaptors with hairpin loop structure were ligated to DNA fragments. Size selection was performed with AMPureXP beads (Beckman Coulter, Beverly, USA). Then PCR was performed with Phusion Hi-Fi DNA polymerase, Universal PCR primers and Index (X) Primer. At last, PCR products were purified (AMPure XP system) and library quality was assessed on the Agilent Bioanalyzer 2100 system. The libraries were sequenced on Illumina NovaSeq TM 6000 platform to generate 150 bp paired-end reads, according to the manufacturer’s instructions. Raw gene counts were obtained from the company-provided matrix. To evaluate the transcriptional impact of IBA57 deficiency, we curated two functional gene sets: (i) Fe-S cluster assembly (*IBA57, FDX2, ISCA1, ISCA2, BOLA3, NFU1*) and (ii) representative OXPHOS subunits (*NDUFS1, SDHB, UQCRFS1, COX10, ATP5F1A*). Counts were normalized to counts per million (CPM) and log2-transformed [log2(CPM+1)]. Differential expression of *IBA57* between mutant (fetal) and wild-type (n = 3) samples was assessed by two-tailed Student’s t-test. Expression patterns of the full gene set were displayed as a Z-score-normalized heatmap with raw count annotations. All analyses were performed in Python (v3.11) using pandas, numpy, scipy, matplotlib, and seaborn.

#### 2.6.1 Enrichment analysis of differentially expressed genes

Gene Ontology (GO) enrichment analysis of differentially expressed genes was implemented by the clusterProfiler R package, in which gene length bias was corrected. GO terms with Pvalue less than 0.05 were considered significantly enriched by differential expressed genes. KEGG is a database resource for understanding high-level functions and utilities of the biological system, such as the cell, the organism and the ecosystem, from molecular-level information, especially large-scale molecular datasets generated by genome sequencing and other high-through put experimental technologies (http://www.genome.jp/kegg/). We used clusterProfiler R package to test the statistical enrichment of differential expression genes in KEGG pathways.

### 2.7 LC-MS/MS and metabolite array technology

Amniotic fluid samples were collected via amniocentesis at 21 weeks of gestation in the Department of Obstetrics and Gynecology. Approximately 15–20 mL of amniotic fluid was procured from each participant and subsequently centrifuged at 3000 rpm for 5 min at 4 °C. The cell-free portion of the amniotic fluid was isolated for analysis using LC-MS/MS techniques. Following culturing, the amniotic fluid cells were subjected to Metabolite Array Technology by Maitehuipu Company (Shanghai, China). All fetuses in the control group had normal prenatal diagnostic findings and showed no postnatal complications during follow-up (6 months to 1 year after delivery).

### 2.8 Mitochondrial respiratory chain enzyme activity assays

Fetal liver tissue was obtained by intrauterine biopsy, with a sample size of approximately 50 mg. Immediately after collection, the tissue was snap-frozen in liquid nitrogen and transported on dry ice to MILS (Beijing) Medical Laboratory for mitochondrial respiratory chain enzyme activity assays. The activities of respiratory chain complexes I (NADH:ubiquinone oxidoreductase), II (succinate:ubiquinone oxidoreductase), III (ubiquinol:cytochrome c oxidoreductase), IV (cytochrome c oxidase), and citrate synthase (CS) were measured spectrophotometrically at 30 °C according to established protocols (Kirby et al., 1999; Murayama et al., 2009).

## 3 Results

### 3.1 Clinical and neuroradiological characteristics of the patients

**FIGURE 1:**
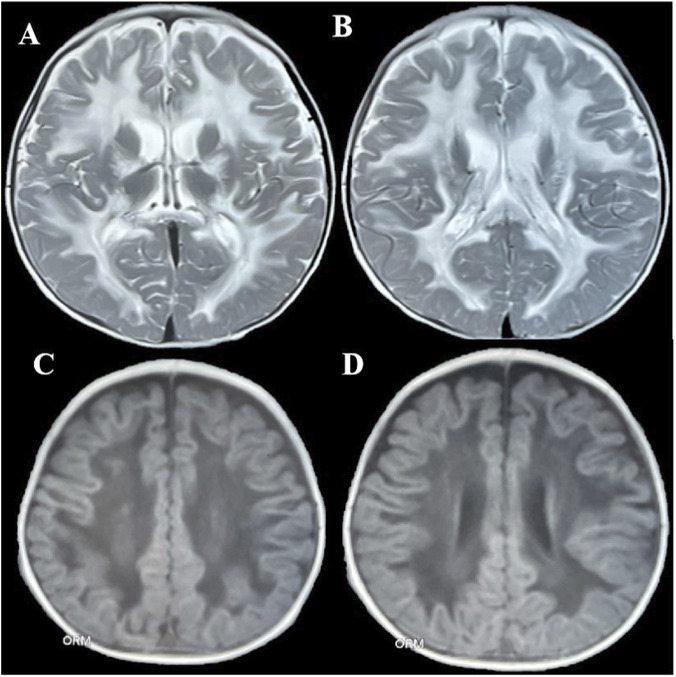
Brain MRI of the patients. **(A,B)** T2-weighted image of proband; **(C,D)** T1-weighted.

Our current study presents the cases of a Chinese male infant and a fetus, both diagnosed with compound heterozygote mutations in the *IBA57*. The proband’s clinical evolution, alongside the prenatal diagnostic work-up in a subsequent pregnancy, is illustrated as a chronological timeline in Supplementary Figure S1. A male infant was delivered vaginally at 40+1 weeks of gestation with a birth weight of 3.28 kg. The infant was normal at birth with no neonatal asphyxia. Non-consanguineous parents following an uneventful pregnancy. Family history was unremarkable. At 2 months of age,the parents noted that the infant could not visually follow objects, which prompted neurological evaluation. However, abnormal periventricular white matter signals were observed on brain magnetic resonance imaging (MRI) performed at 2 months of age. Corresponding axial T2 MRI examination showed symmetrical, confluent T2 hyperintensities in the periventricular white matter of the bilateral fronto-parieto-occipital lobes and cerebellar hemispheres, accompanied by diffuse cerebral atrophy (Figure 1). Physical examination at that time was notable for truncal hypotonia, marked head lag, poor visual tracking, and reduced spontaneous movements; deep tendon reflexes were present but diminished. His diagnosis was “leukoencephalopathy.” Abdominal ultrasound revealed no hepatosplenomegaly, and echocardiography confirmed normal cardiac structure and function. On neurological examination, the infant exhibited spastic tetraparesis, hyperreflexia, sustained ankle clonus, bilateral extensor plantar responses (Babinski sign), minimal social smiling, convergent strabismus. A reexamination of the head MRI((13 months of age) revealed multiple abnormal signals in the bilateral cerebral hemispheres, bilateral cerebellar dentate nuclei, brainstem, corpus callosum, and bilateral posterior limbs of the internal capsule, with deep white matter microcystic degeneration in the left frontal lobe. Laboratory investigations demonstrated a wide-gap metabolic acidosis (anion gap 18 mmol/L) with lactic acidosis (blood lactate 2.4 mmol/L; normal <2.2 mmol/L). Blood gas analysis showed a mixed acid-base disturbance (pCO_2_ 4.67 kPa, pO_2_7.42 kPa). Plasma amino acid profiling revealed decreased ornithine (17.8 µmol/L) and hydroxyproline (19.9 µmol/L), while acylcarnitine analysis demonstrated multiple elevated species including C4-OH (1.720 µmol/L), C5 (0.020 µmol/L), and C14:2 (0.070 µmol/L) (see Supplementary Table S2 for the complete metabolite panel). At 13 months of age, the patient developed a rapidly progressive respiratory infection that necessitated intubation and mechanical ventilation. Sputum culture grew *Streptococcus* pneumoniae (2+) and *Neisseria* species (2+), and he was unable to tolerate oral intake. He died of refractory respiratory failure 4 days after symptom onset.

In the subsequent pregnancy, during the 12+ weeks of gestation, the ultrasound revealed a normal nuchal translucency measurement of 2.7 mm (normal range <3.0 mm). At 21+ weeks of gestation, amniotic fluid samples were obtained at our hospital. Whole exome sequencing (WES) of the family confirmed that the fetus had complex heterozygote mutations in the *IBA57*, as validated by Sanger sequencing. No structural abnormalities were detected during an ultrasound examination. Analysis of the amniotic fluid using a validated LC-MS/MS method and GC-MS analysis revealed elevated levels of Ornithine (184.75 µmol/L) and C4DC (0.16 µmol/L), while Glucose1, Glucose2, sucrose, myo-inositol, lactose, 4HPP (4-hydroxyphenylpyruvic acid), and Urate showed slight increases (Supplementary Figure S2).

### 3.2 Genetic findings

**FIGURE 2:**
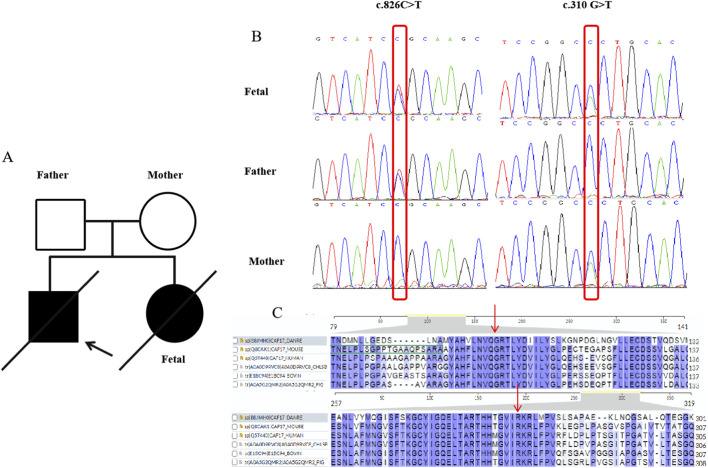
Pedigree and variants. **(A)** Pedigree of affected individual, the proband was diagnosed with Biallelic variants in *IBA57* through Whole Exome Sequencing (WES) and confirmation by Sanger sequencing at other hospital prior to their passing. **(B)** Sanger sequencing validation of *IBA57* c.826C>T (forward strand, C>T) and c.310G>T (reverse strand, C>A). **(C)** Multispecies sequence alignment of the IBA57 protein showing the evolutionary conservation of the amino acid at G104 and R276. The conserved G104 and R276 residues are indicated by the red arrow.The species from top to bottom are: Zebrafish, Mouse,Human, Monkey,Bovin,PIG.

As a result, no pathogenic mitochondrial variants were identified and CMA find no pathogenic duplication or deletion in the whole family. A single gene entry corresponding to *IBA57* (NM_001010867.3; NP_001010867.1) was identified for both patients. Based on the ACMG guidelines, the c.310G>T (p.Gly104Cys) variant is classified with the following evidence criteria: PM2_Supporting + PM3_moderate + PP3_Strong + PP4. The *IBA57* c.826C>T (p.Arg276Cys) variant is classified as: PM2_Supporting + PM3_Strong + PP3_Moderate + PP4. Detailed supporting evidence, including population frequency, segregation, evolutionary conservation, and *in silico* predictions, is provided in Supplementary Table S1. These variants were transmitted from their heterozygous father and mother, respectively (Figure 2B). All variants in our study were highly conserva-tive in the related species (Figure 2C).

### 3.3 Histological examination

**FIGURE 3:**
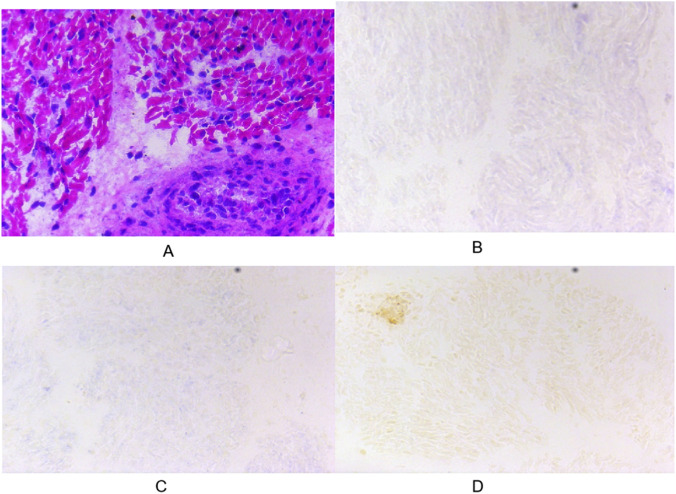
Pathological picture. **(A)** HE-stained. **(B)** Nicotinamide adenine dinucleotide (NADH) staining showed an absence of muscle fiber enzyme activity. **(C)** The succinate dehydrogenase (SDH) staining indicated loss of muscle fiber enzyme activity. **(D)** Cytochrome-C-Oxidase (COX) staining revealed an absence of muscle fiber enzyme activity.

**FIGURE 4:**
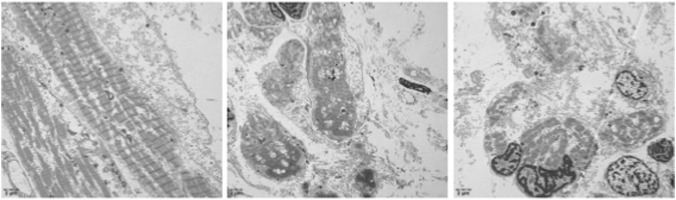
Electron microscopy Muscle fiber myofibrils displaying disorganized arrangement, partial tearing, dissolution, and loss of sarcomeres. Contraction and wrinkling of the sarcolemma. Occasional nuclear migration within muscle cells.Interstitial space showing uneven muscle cell sizes, increased intercellular gaps, and localized collagen fiber proliferation. No significant infiltration of inflammatory cells observed. Mild thickening of the capillary basement membrane.

A biopsy was taken from the left gluteus maximus muscle of the 21-week-old fetus, resulting in a small muscle fragment. Microscopic examination revealed skeletal muscle edema, with no significant interstitial hyperplasia observed. Sporadic inflammatory cells were found infiltrating the fascicular membrane and muscle fiber space. The muscle fibers appeared juvenile and had a small diameter of approximately 5–10 μm. Hypertrophy of myofibrous nuclei was observed, appearing oval or short rod-shaped. Diffuse edema, rupture, and dissolution changes were observed in the muscle fibers. Intermuscular nerve bundle structures were visible (Figure 3A). Modified Gomori Trichrome (MGT) staining did not reveal ragged red fibers or border vacuoles. Nicotinamide adenine dinucleotide (NADH) staining showed an absence of muscle fiber enzyme activity (Figure 4B). The succinate dehydrogenase (SDH) staining indicated loss of muscle fiber enzyme activity (Figure 4C). Cytochrome-C-Oxidase(COX)staining revealed an absence of muscle fiber enzyme activity (Figure 4D).

Electron microscopic examination revealed pathological changes in the muscle tissue. The arrangement of myocyte myofibrils appeared disordered, with some myofibrils showing signs of tearing, dissolution, and disappearance of segments. There was shrinkage observed in the myofilaments. No proliferation or aggregation of muscle nuclei was observed. Myonuclear migration was rare, and axial voids were not seen. There was no significant increase observed in mitochondria, glycogen, or lipid droplets. Rod-shaped bodies, inclusion bodies, and tube aggregations were not observed. In the intermuscular substance, there was uneven cell size, widened gaps, local collagen fiber hyperplasia, and no significant infiltration of inflammatory cells. The capillary basement membrane showed slight thickening (Figure 4).

### 3.4 Western blotting analysis

**FIGURE 5:**
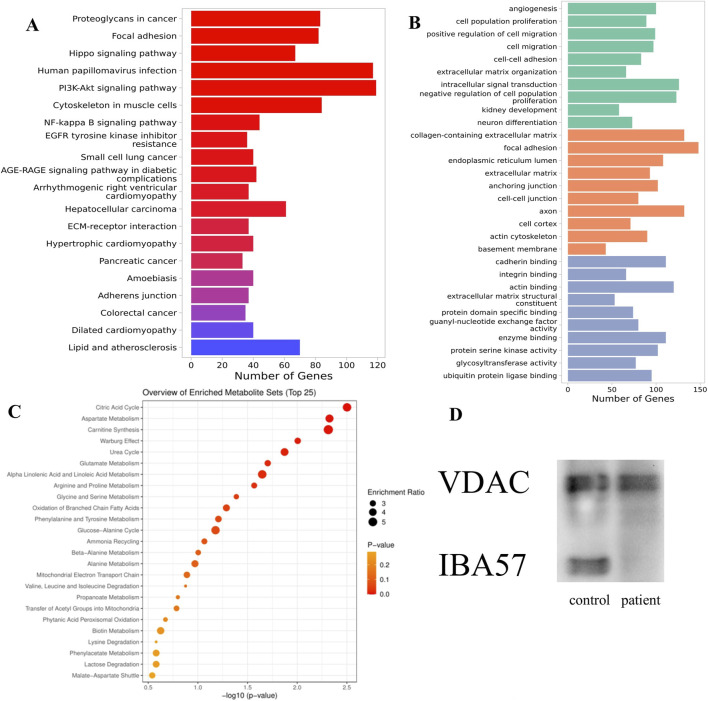
Differential expression and enriched pathway analysis. **(A)** The bar plot presented the enrichment scores (−loge[p value]) of the top 20 significantly enriched GO terms in biological processes, cellular components and molecular functions, Y-axis showed the name of enriched pathways. **(B)** KEGG analysis for the host genes of differentially expressed RNA. Rich factor represented the enrichment degree of differentially expressed genes. **(C)** Represents a metabolomics analysis graph. **(D)** IBA57 in amniotic fluid cells.

Western blot analysis was conducted on mitochondria extracted from amniotic fluid samples. The results showed a significant decrease in *IBA57* expression in the 21-week fetal sample compared to control cells obtained from a normal amniotic fluid sample at 21+3 weeks, due to advanced maternal age (>35 years old) (Figure 5D).

### 3.5 Transcriptomic profiling and functional validation in *IBA57*-mutant amniocytes

Transcription patterns were analyzed in amniocyte samples from a single affected fetus carrying *IBA57* compound heterozygous mutations(3 repetitions)and three independent normal controls (fetuses with normal postnatal development). The analysis of transcriptomic revealed distinct clustering of transcription levels between the patient and the normal samples. Further analysis was conducted to identify differentially expressed genes between *IBA57*-mut and Wild-type (p < 0.05, |Log2 Fold Change|>2) (Supplementary Figure S3).

GO and KEGG analyses were performed to investigate the functions of differentially expressed RNA and explore molecular interactions among genes. The results of the KEGG pathway analysis are presented in Figure 5A. The genes of the differentially expressed RNA were found to be predominantly associated with pathways such as the PI3K-Akt signaling pathway. The Gene Ontology (GO) consists of three domains: cellular component, which refers to the various parts of a cell and the extracellular environment; molecular function, which describes the main activities of gene products at the molecular level; and biological processes, which are events or actions that occur within a cell, defining a beginning and an end. The top 10 enriched GO terms in biological process, cellular component, and molecular function are depicted in Figure 5B.

**FIGURE 6:**
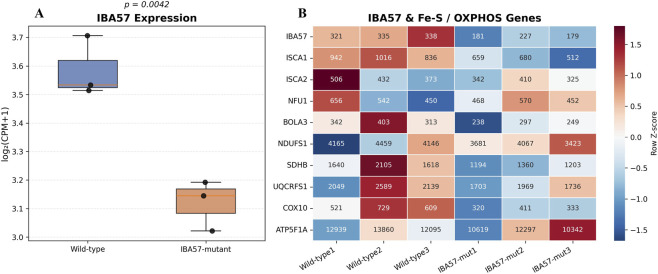
IBA57 deficiency disrupts the coordinated expression of Fe-S cluster assembly and OXPHOS genes. **(A)** *IBA57* expression is significantly reduced in amniocyte. Boxplots show normalized expression levels [log2(CPM+1)]; the p-value was calculated by two-tailed Student’s t-test. **(B)** Heatmap of row-wise Z-scores for the curated Fe-S cluster and OXPHOS gene sets. Numbers inside the cells indicate raw counts. WT, wild-type; Mut, *IBA57*-mutant.

The box plot in Figure 6A demonstrated that *IBA57* expression was significantly decreased in the *IBA57*-mutant group compared with the wild-type control (p = 0.0042). The heatmap further revealed a coordinated downregulation of genes involved in Fe-S cluster assembly (*IBA57, FDX2, ISCA1, ISCA2, BOLA3, NFU1*) and oxidative phosphorylation (*NDUFS1, SDHB, UQCRFS1, COX10, ATP5F1A*) in the mutant sample (Figure 6B). To determine whether the transcriptional downregulation of Fe-S cluster and OXPHOS genes was accompanied by functional defects at the enzymatic level, we measured the activities of respiratory chain complexes I-IV in fetal liver homogenates. Complex II activity in the affected fetus was 20.1% of the normal control mean. The complex II/CS ratio was 38.2%. Complexes I, III, and IV activities were within normal limits (Supplementary Table S3).

### 3.6 Metabolite array test

We employed a novel methodology for simultaneous analysis of metabolites, which have been demonstrated to effectively distinguish between disease and healthy controls in numerous studies. The identified metabolites included bile acids, amino acids, organic acids, indole derivatives, phenolic compounds, and phenyl derivatives (Xie et al., 2021). This study represents the first instance in which metabolite array testing has been conducted on amniocytes. It is found that metabolites expressed in mitochondria, including Oleylcarnitine, N-Acetyaspartic acid, Hexanylcarnitine, EPA(eicosapentaenoic acid), Valerylcarnitine, and DPA(docosapentaenoic acid), Linoleylcarnitine exhibited unusually high levels. Glucose 6-Phosphate and Fructose 6-Phosphate are decreased. We performed enrichment analysis on the top 25 altered metabolite sets (Figure 5C). The most significantly enriched pathway was the citric acid cycle (TCA cycle), followed by coordinated disruptions in aspartate metabolism, carnitine synthesis, the Warburg effect, the urea cycle, and glutamate metabolism. These multiple pathways are all closely associated with mitochondrial energy metabolism.

## 4 Discussion

In this study, we identified compound heterozygous variants in *IBA57*-c.310G>T (p.Gly104Cys) and c.826C>T (p.Arg276Cys) (NM_001010867.4)-in two affected siblings from a Chinese family. Pathogenic mutations in *IBA57* have been linked to MMDS3, a condition characterized by psychomotor regression, optic atrophy, nonspecific dysmorphism, and highly heterogeneous clinical spectrum of mitochondrial diseases. As the disease progresses, symptoms such as encephalopathy, myopathy, vision impairment, seizures, and respiratory failure can arise. It has been observed that there may be symptomatic fluctuations with transient periods of stability, or progression to severe phenotypes, which are often associated with early fatal or debilitating conditions (Zhan et al., 2022). Currently, no disease-specific care guidelines exist for MMDS3 due to its extreme rarity. Management remains largely supportive, guided by general mitochondrial disease consensus recommendations (Parikh et al., 2017). About 32 *IBA57* mutations have been identified, including missense, nonsense, and frame-shift variants (Ajit Bolar et al., 2013; Debray et al., 2015; Hamanaka et al., 2018; Ishiyama et al., 2017; Liu et al., 2018; Torraco et al., 2017) with the majority being loss-of-function mutations. Homozygous variant in *IBA57* (NM_001010867.2): c.310G>T (p.Gly104Cys) was reported by Lang et al. (2022). Sato et al. reported compound heterozygous mutations in the *IBA57* gene (c.49_67dup and c.310G>T) (Sato et al., 2021)). Forny et al. reported a case of mitochondrial leukodystrophy associated with compound heterozygous mutations in the *IBA57* gene (c.802C>T and c.826C>T) (Forny et al., 2021). Specifically, the proband and the affected fetus carried the same compound heterozygous variants in *IBA57*: c.310 G>T and c.826C>T. Functional assessments were conducted using amniotic fluid cells, revealing abnormal expression of *IBA57* impacting fetal neurodevelopment during prenatal verification for the first time. These results expand the known mutational and clinical spectrum of MMDS3 and highlight the utility of multi-omics approaches in rare disease diagnostics.

*IBA57* is located at 1q42.13 and encodes a mitochondrial Fe-S protein assembly factor that adopts a rigid ring-like structure formed by three tightly packed domains. Upon binding a [2Fe-2S] cluster, it forms a heterodimeric complex with ISCA1 and ISCA2 (Gourdoupis et al., 2018; Nasta et al., 2019). IBA57 plays a vital role in the maturation of mitochondrial [4Fe-4S] proteins by facilitating the conversion of [2Fe-2S] to [4Fe-4S] clusters on the scaffold protein Isu1, in concert with the A-type ISC proteins ISCA1 and ISCA2 (Ajit Bolar et al., 2013). The [4Fe-4S] clusters are subsequently inserted into key enzymes of the tricarboxylic acid (TCA) cycle, such as aconitase and succinate dehydrogenase (SDH; complex II), and respiratory chain complexes I–III (Rowe et al., 2020; Rouault, 2012; Tsvetkov et al., 2022). Although respiratory chain complexes I- III all depend on Fe-S clusters, their differential vulnerability to IBA57 deficiency may reflect distinct requirements for the late-acting ISC assembly machinery. The SDHB subunit of complex II is particularly sensitive to IBA57 deficiency. In the affected fetus, complex II activity in liver was reduced to 20.1% of the normal control mean (complex II/CS ratio 38.2%), while complexes I, III, and IV remained within normal limits (Supplementary Table S3). Consistently, SDHB mRNA was markedly downregulated (Figure 6B). These results are in agreement with previous observations that *IBA57* mutations primarily affect SDHB expression without severe reductions in the subunits of complexes I and III (Ishiyama et al., 2017). Thus, the selective loss of complex II activity indicates that IBA57 plays a critical role in SDHB biogenesis and complex II function. IBA57 deficiency compromises TCA cycle function not only directly via impaired aconitase and SDH function but also indirectly by impairing protein lipoylation catalyzed by the mitochondrial [4Fe-4S] protein lipoic acid synthase (Liu et al., 2021). NAD+, a central cofactor of the TCA cycle, participates in numerous biological processes including ATP production, mitochondrial homeostasis, and cell survival (Chan et al., 2012; Lautrup et al., 2019). Notably, NAD + has been reported to interact with [4Fe-4S] cluster-containing proteins (Feng et al., 2022) and to modulate the proliferation and neuronal differentiation of neural stem/progenitor cells (Huang et al., 2022). The severe neurological impairment observed in MMDS3 patients likely results from the convergence of disrupted mitochondrial Fe-S protein maturation, TCA cycle dysfunction, and impaired energy metabolism in neural cells. This mechanistic connection is further supported by the structural damage to cristae membranes described below. Although the present study did not directly assess neural tissues, the coordinated transcriptional and enzymatic defects identified in amniocytes suggest that similar mitochondrial impairments may operate in the developing brain. Future studies using neural-specific models will be required to dissect the precise pathogenic mechanisms.

Notably, previous work has demonstrated that depletion of IBA57, ISCA1, or ISCA2 results in a near-complete loss of cristae membranes and massive mitochondrial enlargement (Sheftel et al., 2010), indicating that IBA57 deficiency compromises mitochondrial inner membrane integrity. Mitochondrial membrane damage is a well-established trigger of cellular energy failure and oxidative stress, which can further amplify organelle dysfunction (Sorrentino et al., 2018). Among cell types, neurons are particularly vulnerable to mitochondrial dysfunction due to their exceptionally high energy demands, and primary mitochondrial disorders typically manifest with prominent neurological deficits including leukoencephalopathy (Chan, 2020). The loss of cristae membranes directly impairs the assembly and stability of OXPHOS complexes, which are densely packed along the cristae and are essential for aerobic ATP production (Chan, 2020). This structural-functional relationship provides a mechanistic basis for the severe white matter degeneration observed in our proband, as oligodendrocytes and neurons require sustained OXPHOS activity for myelination and axonal maintenance.

RNA-seq analysis of amniotic fluid cells with aberrant *IBA57* expression revealed transcriptional alterations in genes associated with the PI3K-Akt signaling pathway; concurrent transcriptional changes in TCA cycle-related genes were also noted. The TCA cycle and PI3K-Akt signaling pathway are interconnected through metabolites like ɑ-ketoglutarate, which influence cellular proliferation and differentiation via pathways regulated by enzymes such as IDH1/2 and GPCRs like OXGR1 (Carbonneau et al., 2016; Wang et al., 2019), although the precise interaction mechanism remains unclear.

Metabolomic profiling of the affected fetal amniocytes revealed disturbed TCA cycle and altered mitochondrial energy metabolism. The observation are in line with metabolic signatures described in IBA57 deficiency (Wongkittichote P et al., 2023). Moreover, the proband presented with hyperlactatemia, which aligns with the metabolic profiles reported in Leigh syndrome (Yoon et al., 2022)and other mitochondrial disorders, where combined abnormalities in lactate, TCA cycle intermediates, and lipid species are common (Clarke C et al., 2013; Correia SP et al., 2024).

Our study has several limitations. The RNA-seq and metabolomic analyses were performed on amniocyte samples from a single affected fetus (the proband’s sibling). As *IBA57* compound heterozygous mutations cause a rare disease, obtaining biological replicates from independent individuals carrying the same mutations is extremely challenging. Although three independent control samples were used to establish a reference baseline, the absence of case replicates precludes statistical comparisons and makes it difficult to exclude individual variability. To partially mitigate this limitation, our findings were cross-referenced with the proband’s clinical metabolic abnormalities and with known pathways linked to IBA57 deficiency. Despite these constraints, the multi-omics data provide valuable insights that complement the clinical and genetic characterization of this rare condition.

## 5 Conclusion

In this study, we investigated a Chinese family in which two siblings—the proband and a subsequent fetus—carried the same compound heterozygous *IBA57* mutations (c.310G>T and c.826C>T), leading to the diagnosis of MMDS3.

Integrated transcriptomic and metabolomic analyses, combined with respiratory chain enzyme activity assays in *IBA57*-mutant amniocytes, not only yielded results consistent with the metabolic signature of IBA57 deficiency, but also identified distinctive metabolic features of the disease through comparison with other mitochondrial disorders, thereby broadening the disease spectrum and phenotypic spectrum of MMDS3.

## Data Availability

The datasets for this article are not publicly available due to concerns regarding participant/patient anonymity. Request to access the datasets should be directed to the corresponding authors.
