# Supplementary material for: Biallelic variants in IBA57 with multiple mitochondrial dysfunction syndrome 3
Source: Front Genet. 2026 Jul 20;17:1815601. doi: 10.3389/fgene.2026.1815601 (PMC13429234; doi:10.3389/fgene.2026.1815601)
Supplement: Supplementary file 1 [file DataSheet1.docx]

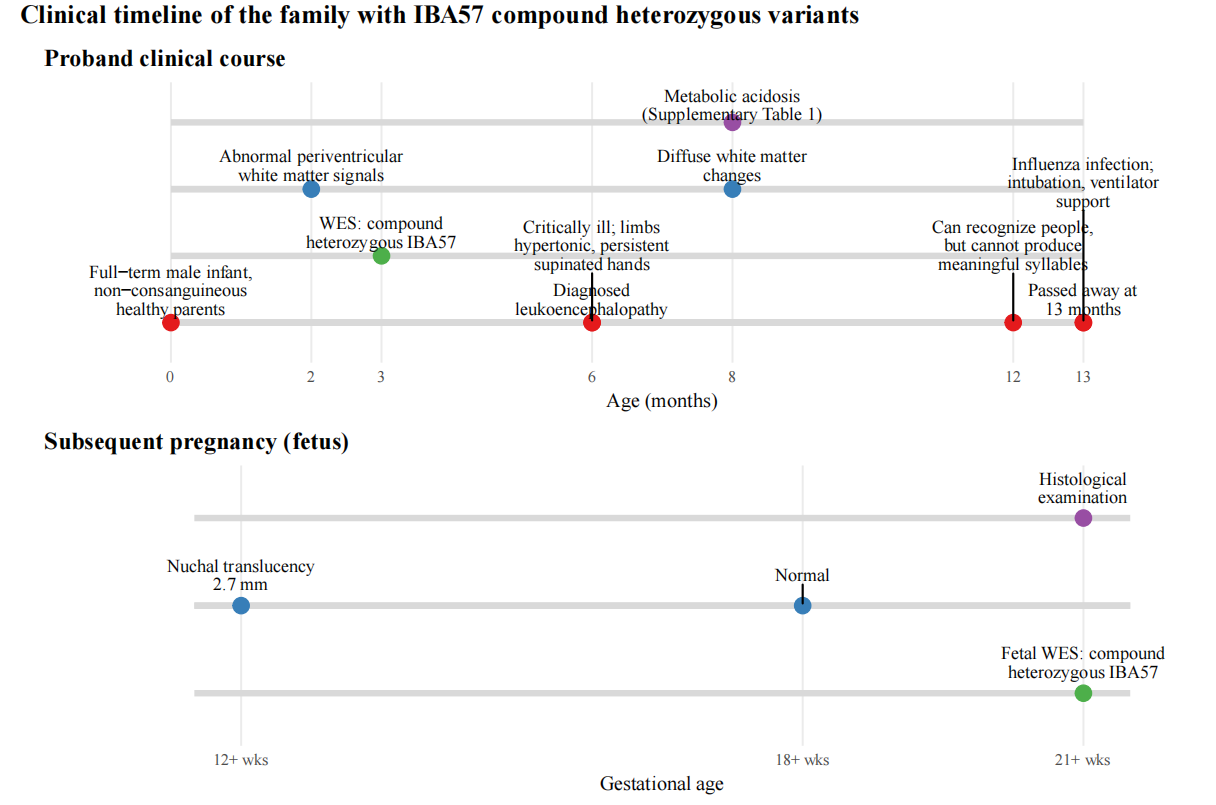


**Figure 1. Clinical timeline of the family with *IBA57* compound heterozygous variants.** Timeline of key clinical, imaging, laboratory, and genetic events for the proband (upper panel) and the subsequent pregnancy (lower panel). The proband’s clinical course spans from birth to death at 13 months of age, including brain MRI findings, genetic diagnosis, physical examination, metabolic investigations, and terminal deterioration following influenza infection. The lower panel depicts prenatal investigations in a subsequent pregnancy, including nuchal translucency ultrasound, fetal anatomy scan, amniocentesis with whole-exome sequencing, and histological examination of fetal tissue. Abbreviations: WES, whole-exome sequencing; NT, nuchal translucency.


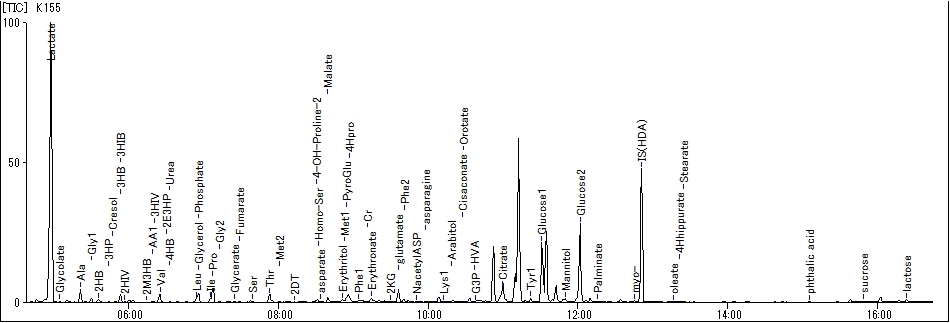


**Figure 2: Targeted metabolomic analysis of amniotic fluid.** Using a validated LC-MS/MS method, ornithine (184.75µmol/L; reference range 15-50µmol/L) and C4DC (0.16µmol/L; reference range 0.04-0.12 µmol/L) were markedly elevated. Compared with the normal control group, the patient sample showed the following fold changes: glucose1, 2.36; glucose2, 2.37; sucrose, 3.23; myo-inositol, 4.79; and lactose, 2.83. 4HPP (4-hydroxyphenylpyruvic acid) and urate were undetectable in control amniotic fluid but were present in the patient sample at peak areas of 1,540 and 5,965, respectively.

| Gene | cDNA change | Allele frequency in gnomAD (Aggregated) | Segregation evidence | Evolutionary conservation | Revel score | SIFT &PolyPhen-2 |
| --- | --- | --- | --- | --- | --- | --- |
| *IBA57* | c.310G>T | No Observation | Heterozygous in unaffected parents(Mother) | GERP  Uncertain (4.32) | 0.95(Strong) | Deleterious (Supporting) (0) & (N/A) |
| *IBA57* | c.826C>T | 0.0116%(Very Rare ) | Heterozygous in unaffected parents(Father) | GERP  Uncertain (4.12) | 0.81(Moderate) | Deleterious (Supporting) (0) & Deleterious (Moderate) (1) |

**Table 1. Supporting evidence for the classification of IBA57 variants.** Population frequency, segregation, evolutionary conservation, GERP conservation scores, and in silico predictions (REVEL, SIFT, PolyPhen‑2). For the SIFT & PolyPhen‑2 column, values in parentheses are the SIFT score (0 = deleterious) and the PolyPhen‑2 HumVar score (1 = probably damaging). N/A, not available.

| Project | Value | Reference ranges |
| --- | --- | --- |
| pH | 7.049 | 7.31~7.41 |
| PCO_2_ | 4.67kPa | 5.3~6.7 kPa |
| PO_2_ | 7.42kPa | 4.0~5.3 kPa |
| LA | 2.4mmol/L | <2.2 mmol/L |
| AG | 18mmol/L | 5~15 mmol/L |
| CHO | 5.42mmol/L | <5.2 mmol/L |
| HDL | 0.75mmol/L | ≥1.0 mmol/L |
| LDL | 4.62mmol/L | <3.4 mmol/L |
| GLU | 5.82mmol/L | 3.3~5.6 mmol/L |
| C4-OH | 1.720µmol/L | <0.50 µmol/L |
| C5 | 10.020µmol/L | <0.50 µmol/L |
| C14 | 20.70µmol/L | <0.60 µmol/L |
| (C3DC+C4-OH)/C10 | 15.42 | <0.20~0.25 |
| C4/C3 | 0.52 | <0.30 |
| C5/C3 | 0.16 | <0.03 |
| C5DC/C10 | 0.09 | <0.08 |
| C5-OH/C8 | 0.72 | <0.5 |
| C5-OH/C16 | 0.4 | <0.05~0.10 |
| C8/C10 | 0.84 | <1.0 |
| C10:2/C19 | 0.19 | <0.05~0.10 |
| C14:1/C14 | 3.2 | <1.5 |
| Orn | 17.8µmol/L | 20~130 µmol/L |
| Hyp | 19.9µmol/L | 10~45 µmol/L |
| beta-galactosidase | 155.8nmol/mg.h | 97~367 nmol/mg.h |
| ARSA | 372.3nmol/mg | 100~500 nmol/mg.h |

**Table 2. Using validated LC-MS/MS method analysis blood reveal metabolic acidosis and All metabolites in the list are elevated.**


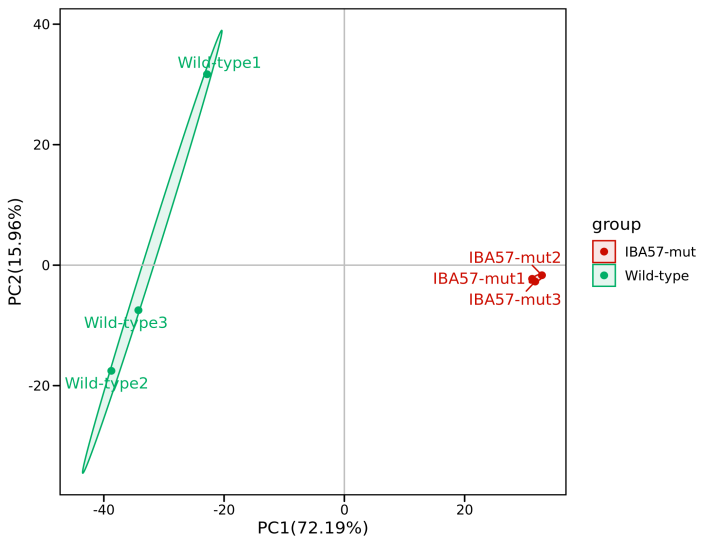

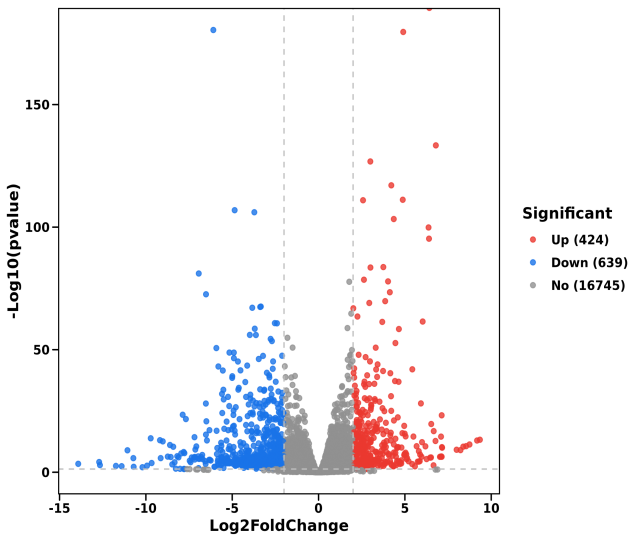


A B B

**Figure 3** **RNA sequencing** A In the 2D PCA analysis, a scatter plot is displayed showing transcription patterns analyzed in amniocyte samples from a single affected fetus carrying *IBA57* compound heterozygous mutations (*IBA57*-mut) and three independent normal controls (Wild-type), each point representing one sample. B The horizontal axis represents the log2 fold change(p<0.05, |Log2 Fold Change|>2) in gene expression across different samples or comparison groups, with a larger absolute value indicating a greater fold change in expression the two conditions. The vertical axis represents the significance level of the expression difference. Genes/transcripts with upregulated expression are indicated by red dots, while those with downregulated expression are indicated by blue dots.

|  | **Co I** | **Co II** | **Co III** | **Co IV** | **CS** |
| --- | --- | --- | --- | --- | --- |
| **Control** |  |  |  |  |  |
| % of normal | **74.4** | **66.4** | **66.5** | **128.8** | **78.5** |
| CS ratio (%) | **89.9** | **81.1** | **82.2** | **157** |  |
| Co II ratio (%) | **111.2** |  | **96.8** | **167.7** |  |
| **Fetal（Hep1）** |  |  |  |  |  |
| % of normal | **49.1** | **20.1** | **41.3** | **104.5** | **50.4** |
| CS ratio (%) | **92.5** | **38.2** | **79.7** | **198.5** |  |
| Co II ratio (%) | **243.1** |  | **199.3** | **450.6** |  |

**Table 3 Mitochondrial respiratory chain enzyme activities in fetal liver homogenates.** Complex I-IV: respiratory chain complexes I-IV; CS: citrate synthase. “% of normal” represents enzyme activity expressed as a percentage of the control mean. “CS ratio” and “Co II ratio” indicate activity normalized to CS and complex II, respectively.

## **Materials and Methods of Whole-Exome Sequencing**

## Reads were aligned to the human reference genome (GRCh38) using BWA-MEM, and variants were called following GATK best practices. Annotation was performed with SnpEff (Cingolani et al., 2012), ANNOVAR (Wang et al., 2010), and InterVar (Li & Wang et al., 2017). All variants were visually inspected using the Integrative Genomics Viewer (Robinson et al., 2011). For the fetal sample, targeted resequencing was performed using an in‑house implemented pipeline, which incorporated functional annotation via the dbNSFP/WGSA framework and filtered variants against public databases (dbSNP142, 1000 Genomes, UK10K, ExAC) and in‑house population databases. Mitochondrial DNA variants were analyzed from the off-target reads of the whole-exome sequencing data. Reads were aligned to the revised Cambridge Reference Sequence (NC_012920.1). Mitochondrial genome coverage exceeded 3,000×mean depth, with 100% of bases covered at ≥1,000×depth. All variants identified in the affected individuals were annotated with databases such as refGene, Avsnp150, gnomAD211, Clinvar, dbnsfp41a, Intervar by snpeff5.0d and annovar 2020 Jun. Candidate mutational events were then inspected with the integrative genomics viewer (Robinson et al., 2011). Variants with a minor allele frequency greater than 0.01 in the Genome Aggregation Database (gnomAD v2.1.1) were excluded. The candidate variants were classified according to the American College of Medical Genetics and Genomics and the Association for Molecular Pathology (ACMG/AMP) guidelines (Richards et al., 2015).
